# *HEADLESS*, a *WUSCHEL* homolog, uncovers novel aspects of shoot meristem regulation and leaf blade development in *Medicago truncatula*

**DOI:** 10.1093/jxb/ery346

**Published:** 2018-09-29

**Authors:** Yingying Meng, Huan Liu, Hui Wang, Ye Liu, Butuo Zhu, Zuoyi Wang, Yaling Hou, Pengcheng Zhang, Jiangqi Wen, Hongshan Yang, Kirankumar S Mysore, Jianghua Chen, Million Tadege, Lifang Niu, Hao Lin

**Affiliations:** 1 Biotechnology Research Institute, Chinese Academy of Agricultural Sciences, Beijing, China; 2 Department of Plant and Soil Sciences, Institute for Agricultural Biosciences, Oklahoma State University, Sam Noble Parkway, Ardmore, OK, USA; 3 Key Laboratory of Tropical Plant Resources and Sustainable Use, CAS Center for Excellence in Molecular Plant Sciences, Xishuangbanna Tropical Botanical Garden, Chinese Academy of Sciences, Kunming, Yunnan Province, China; 4 Noble Research Institute, LLC, Sam Noble Parkway, Ardmore, OK, USA; 5 Lanzhou Institute of Husbandry and Pharmaceutical Sciences, Chinese Academy of Agricultural Sciences, Lanzhou, Gansu Province, China

**Keywords:** *HEADLESS*, leaf development, *Medicago truncatula*, SAM maintenance, transcriptional repressor, *WUSCHEL* homolog

## Abstract

The formation and maintenance of the shoot apical meristem (SAM) are critical for plant development. However, the underlying molecular mechanism of regulating meristematic cell activity is poorly understood in the model legume *Medicago truncatula*. Using forward genetic approaches, we identified *HEADLESS* (*HDL*), a homolog of Arabidopsis *WUSCHEL*, required for SAM maintenance and leaf development in *M. truncatula*. Disruption of *HDL* led to disorganized specification and arrest of the SAM and axillary meristems, resulting in the *hdl* mutant being locked in the vegetative phase without apparent stem elongation. *hdl* mutant leaves are shorter in the proximal–distal axis due to reduced leaf length elongation, which resulted in a higher blade width/length ratio and altered leaf shape, uncovering novel phenotypes undescribed in the Arabidopsis *wus* mutant. HDL functions as a transcriptional repressor by recruiting MtTPL through its conserved WUS-box and EAR-like motif. Further genetic analysis revealed that *HDL* and *STENOFOLIA* (*STF*), a key regulator of *M. truncatula* lamina outgrowth, act independently in leaf development although HDL could recruit MtTPL in the same manner as STF does. Our results indicate that HDL has conserved and novel functions in regulating shoot meristems and leaf shape in *M. truncatula*, providing new avenues for understanding meristem biology and plant development.

## Teaser

The WOX family transcription factor HEADLESS exhibits a repressive activity and performs conserved and novel functions in the regulation of shoot meristems and leaf shape in *Medicago truncatula*.

## Introduction

The shoot apical meristem (SAM) is made of pluripotent stem cells located at the shoot apex, which is responsible for self-maintenance and producing lateral organ primordia that develop into post-embryonic aerial organs (Barton, 2010). The SAM provides an environment for maintenance of meristematic cell activity and is regarded as a plant stem cell niche (Busch *et al.*, 2010). Several key regulators of stem cell control in the SAM have previously been identified through genetic approaches. The homeobox gene, *WUSCHEL* (*WUS*), which is specifically expressed in the organizing center of the SAM, plays a central role in shoot stem cell identity and is required for non-cell-autonomous induction and maintenance of stem cell fate in Arabidopsis (Laux *et al.*, 1996; Mayer *et al.*, 1998; Lenhard *et al.*, 2001; Yadav *et al.*, 2010). In Arabidopsis *wus-1* mutant plants, the defect is exhibited at all developmental stages, and the shoot stem cells are misspecified, resulting in the premature termination of SAMs and floral meristems, and leading to an aberrant flat morphology (Laux *et al.*, 1996; Mayer *et al.*, 1998). The CLE peptide CLAVATA3 (CLV3) acts as a negative signal for shoot stem cell proliferation (Fletcher *et al.*, 1999; Kondo *et al.*, 2006; Ohyama *et al.*, 2009). Several receptor-like protein kinases, including CLV1, RECEPTOR-LIKE PROTEIN KINASE 2 (RPK2), and CLAVATA3 INSENSITIVE RECEPTOR KINASES (CIKs), the receptor-like protein CLV2, and the pseudokinase CORYNE (CRN) are required for the perception of the CLV3 signal to repress *WUS* expression (Clark *et al.*, 1997; Kayes and Clark, 1998; Müller *et al.*, 2008; Ogawa *et al.*, 2008; Kinoshita *et al.*, 2010; Hu *et al.*, 2018). In turn, *WUS* directly activates *CLV3* expression within the central zone, indicating that SAM maintenance is controlled by a complex *WUSCHEL*–*CLAVATA* feedback loop (Brand *et al.*, 2000; Schoof *et al.*, 2000; Somssich *et al.*, 2016). Recent studies in Arabidopsis using computational and experimental approaches revealed that WUS activates *CLV3* only in the absence of HAIRY MERISTEM (HAM) proteins, and an apical–basal gradient of HAM defines the expression pattern of *CLV3* in the initiating meristems (Zhou *et al.*, 2018).

The phytohormone cytokinin is essential for maintenance of the shoot meristem activity and cell proliferation (Riou-Khamlichi *et al.*, 1999; Werner *et al.*, 2003). It has been shown that the WUS protein is linked to cytokinin signaling through direct transcriptional repression of type-A *Arabidopsis Response Regulator* (*ARR*) genes (Leibfried *et al.*, 2005), which are negative regulators of cytokinin signaling. In Arabidopsis, the expression of the closely related type-A response regulator genes including *ARR*5, *ARR6*, *ARR7*, and *ARR15* is repressed by WUS in the center of the SAM. Ectopic expression of an active phosphorylated form of *ARR7* causes the formation of an aberrant SAM, indicating that WUS controls meristem function by directly regulating cytokinin-inducible response regulators (Leibfried *et al.*, 2005). WUS mainly functions as a transcriptional repressor in SAM maintenance and interacts with TOPLESS (TPL) and TPL-related (TPR) transcriptional co-repressors, which are recruited via their conserved WUS-box (Kieffer *et al.*, 2006; Ikeda *et al.*, 2009; Causier *et al.*, 2012; Dolzblasz *et al.*, 2016).

The plant specific *WUSCHEL*-related homeobox (WOX) family genes, of which *WUS* is the founding member, fulfill specialized functions in several key developmental processes such as embryonic patterning, vascular patterning, and lateral organ development (van der Graaff *et al.*, 2009; Costanzo *et al.*, 2014). It has been reported that WOX transcription factors play essential roles in leaf development in various species, including both monocots and eudicots. In monocots, two maize *WOX3*/*PRS* homologs, *NARROW SHEATH1* and *NARROW SHEATH2* (*NS1* and *NS2*), redundantly regulate leaf blade outgrowth, as demonstrated by the *ns1 ns2* double mutant with a severe narrow leaf phenotype (Scanlon *et al.*, 1996; Nardmann *et al.*, 2004). Similarly, simultaneous mutations in *NS* homologous genes, *NARROW LEAF2* and *NARROW LEAF3* (*NAL2* and *NAL3*), also result in a narrow leaf blade phenotype in rice (Cho *et al.*, 2013; Ishiwata *et al.*, 2013). In eudicots, the Arabidopsis *WOX3*, called *PRESSED FLOWER* (*PRS*), specifically regulates the lateral axis-dependent development of flowers (Matsumoto and Okada, 2001), while combining *prs* with *wox1* in the *wox1 prs* double mutant leads to a narrow leaf blade defect indicating that *WOX1* and *PRS* play functionally redundant roles in regulating leaf blade development in Arabidopsis (Vandenbussche *et al.*, 2009; Nakata *et al.*, 2012). In the model legume *Medicago truncatula* and woodland tobacco (*Nicotiana sylvestris*), loss of function in the *WOX1* homolog *STENOFOLIA* (*STF*) and *LAMINALESS1* (*LAM1*), respectively, is sufficient to arrest lateral leaf blade outgrowth, leading to severe defects in leaf width, as well as vein patterning (Tadege *et al.*, 2011). Similarly, other homologs *MAEWEST* (*MAW*) in petunia and *LATHYROIDES* (*LATH*) in pea are also involved in controlling leaf blade development and petal fusion (Vandenbussche *et al.*, 2009; Zhuang *et al.*, 2012). STF promotes leaf blade outgrowth by activating cell proliferation at the adaxial–abaxial juxtaposition of the leaf margin with a transcriptional repression mechanism (Lin *et al*, 2013; Zhang *et al*, 2014). This repression requires MtTPL in a manner analogous to WUS function in the SAM (Zhang *et al*, 2014). In fact, this function can be substituted by WUS.

Expression of Arabidopsis WUS under control of the *M. truncatula STF* promoter can complement both the *stf* and *lam1* mutant phenotypes, indicating that a WUS-like function might be required for cell proliferation in the determinate leaf blade tissue in *M. truncatula* and *N. sylvestris* (Tadege *et al.*, 2011). *WUS* can also complement the *prs* and *wox5* mutant phenotypes if expressed under the control of appropriate promoters (Sarkar *et al.*, 2007; Shimizu *et al.*, 2009), suggesting a common mechanism in the function of repressive *WOX* genes. Nevertheless, *WOX* genes have very specific and restricted expression patterns, and *WUS* expression outside of the vegetative SAM has been reported in the monocots rice and maize (Nardmann and Werr, 2006; Lu *et al.*, 2015; Tanaka *et al.*, 2015), and recent studies revealed that *WUS* expression could also be activated in the leaf axil to promote axillary meristem initiation in Arabidopsis (J. Wang *et al.*, 2017). Several reports described the genetic manipulation and characterization of *WUS* in several species but, in eudicots, *wus* loss-of-function genetic mutants have so far been reported in Arabidopsis, Petunia, and *Antirrhinum* (Laux *et al.*, 1996; Stuurman *et al.*, 2002; Kieffer *et al.*, 2006), where phenotypes are characterized by a ‘stop-and-go’ type of growth habit (organogenesis and termination are constantly reiterated in mutant plants) with aberrant vegetative and inflorescence meristems (Laux *et al.*, 1996; Stuurman *et al.*, 2002; Kieffer *et al.*, 2006; J. Wang *et al.*, 2017). Importantly, the molecular function of the *WUS* homolog in *M. truncatula* is poorly understood.

Here, we report the isolation and characterization of *headless* (*hdl*) from *M. truncatula*, which is defective in SAM development. We show that *HDL* is the homolog of *WUSCHEL* in Arabidopsis and is required for maintaining shoot meristem activity and leaf shape in *M. truncatula*. Unlike the *wus* mutant, the *hdl* mutant is stemless and never flowers, but, like WUS, HDL exhibits a repressive activity and represses the expression of several type-A response regulators in the *M. truncatula* shoot apex.

## Materials and methods

### Plant materials and growth conditions

*Medicago truncatula* ecotype R108 was used for all experiments described in this study. *hdl-1* (NF11982), *hdl-2* (NF3119), and *hdl-3* (NF2389) alleles were identified from the *Tnt1* retrotransposon-tagged mutant collection of *M. truncatula* (Tadege *et al.*, 2008). Scarified *M. truncatula* seeds were germinated overnight on moist Petri dishes, and placed at 4 °C for 1 week, except for the 12 h germinated seeds for SEM analyses. Plants were grown at 25 °C day/23 °C night temperature, 16 h day/8 h night photoperiod, 60–70% relative humidity, and 150 μmol m^–2^ s^–1^ light intensity.

### Plasmid constructs and plant transformation

To make the complementation construct, the *HDL* genomic DNA containing a 2713 bp upstream sequence, the entire *HDL* gene, and a 1366 bp downstream region was cloned into the binary vector pCAMBIA2300 using HDL-pro-*Kpn*I-iF and HDL-1366ASC-*Pst*I-iR primers (Supplementary Table S1 at *JXB* online). The destination construct was introduced into *Agrobacterium tumefaciens* by chemical transformation. *Agrobacterium tumefaciences* strain AGL1 was used for *M. truncatula* transformation as previously described (Meng *et al.*, 2017).

### RNA extraction and quantitative RT–PCR

Total RNA was extracted from shoot apices or unexpanded young leaf tissues of corresponding *M. truncatula* plants by using TRIzol^®^ reagent (Invitrogen). cDNA was generated by reverse transcription with SuperScript III (Invitrogen). Quantitative reverse transcription–PCR (RT–PCR) was performed as previously described (H. Wang *et al.*, 2017) with at least three biological and three technical replicates for both samples and controls. *MtActin* was used as the internal control. All primers used in this study are listed in Supplementary Table S1.

### Subcellular localization and confocal microscopy

For subcellular localization of HDL, *A. tumefaciens* strain GV2260 containing *pMDC83-HDL* and the nuclear marker plasmid *p35S::mRFP-AHL22* or *pMDC32-GFP* and *p35S::mRFP-AHL22* were simultaneously infiltrated into 3- to 4-week-old *Nicotiana benthamiana* leaves. The *pMDC32-GFP* was used as the control. P19 from *Tomato bushy stunt virus* was used to inhibit transgenic silencing. The fluorescence signal was observed with a confocal microscope (Zeiss LSM700) 48–60 h after infiltration.

### Histological analysis and *in situ* hybridization

Tissues of *M. truncatula* were ﬁxed and embedded as previously described (Lin *et al.*, 2013*a*). The tissues were sliced into 8–10 μm sections with a Leica RM2265 microtome, afﬁxed to microscope slides, and stained with toluidine blue. Images were obtained with a digital camera mounted on the Olympus BX-51 compound microscope.

RNA *in situ* hybridization was performed essentially as described previously (Chen *et al.*, 2012). RNA antisense and sense probes were generated with the T7 and SP6 polymerases, respectively, using a 909 bp HDL cDNA template. Sections from shoot apices of 4-week-old wild-type R108 plants were processed and hybridized with digoxigenin-labeled sense and antisense probes. Slides were observed under bright field through an Olympus BX63 microscope.

### SEM analysis

For SEM, fresh *M. truncatula* shoot tissues were fixed with 3.0% glutaraldehyde in 25 mM phosphate buffer (pH 7.0) for 2 d, then plant tissues were further fixed with 1.0% osmium tetroxide in 25 mM phosphate buffer for 2 h and subsequently dehydrated in a graded ethanol series. The desiccated tissues were critical-point dried in liquid CO_2_, mounted on aluminum stubs, and sputter coated with gold. Specimens were then observed using a JSM-8404 microscope (S3400N; Hitachi Ltd).

### Y2H and BiFC assays

The yeast two-hybrid (Y2H) assay was performed according to the manufacturer’s instructions (ProQuest two-hybrid system with Gateway technology; Invitrogen). The mutations in *HDL* (*HDLm1*, *HDLm2*, *HDLm1m2*, *HDLmAD*, *HDLmADm1*, *HDLmADm2*, and *HDLmADm1m2*) were introduced by PCR site-directed mutagenesis using appropriate primers as previously described (Lin *et al.*, 2013*a*). For Y2H assay, the coding sequences of *HDL* and the mutant derivatives of *HDL*, *HDLm1*, *HDLm2*, *HDLm1m2*, *HDLmAD*, *HDLmADm1*, *HDLmADm2*, and *HDLmADm1m2* were cloned into pGADT7-GW (AD), and the coding sequences of *MtTPL* were cloned into pGBKT7-GW (BD) using the Gateway system (Invitrogen). The bait and prey plasmids were co-transformed into the Y2H gold yeast strain (Clontech). For the auxotrophic assay, yeast colonies were patched onto SD/-Leu/-Trp (DDO) and SD/-Trp/-Leu/-His/-Ade (QDO) plates, and grown in darkness at 28 °C for 3 d.

Bimolecular fluorescence complementation (BiFC) assays were conducted as described (Lu *et al.*, 2010). Briefly, *HDL*, *HDLm1*, *HDLm2*, *HDLm1m2*, *HDLmAD*, *HDLmADm1*, *HDLmADm2*, and *HDLmADm1m2* were cloned into pEARLEYGATE201-YN, while *MtTPL* and *HDL* were cloned into pEARLEYGATE202-YC by the LR reaction. HDL-YN, HDLm1-YN, HDLm2-YN, HDLm1m2-YN, HDLmAD-YN, HDLmADm1-YN, HDLmADm2-YN, HDLmADm1m2-YN, and MtTPL-YC, as well as HDL-YN and HDL-YC were introduced into *A. tumefaciens* strain GV2260. Combinations of plasmids were simultaneously infiltrated into 3- to 4-week-old *N. benthamiana* leaves. P19 was used to inhibit transgenic silencing. The fluorescence signal was observed with a confocal microscope (Zeiss LSM700) 48–60 h after infiltration.

### Transient luciferase expression assay

Construction of the reporter GAL4-LUC plasmid was described previously (Lin *et al.*, 2013*a*). For effector plasmids, the coding sequences of *HDL* or *HDL-VP64* were first cloned into pGBKT7. Then the coding regions of the BD fusion were amplified using specific primers and cloned into p2GW7 using the Gateway system (Invitrogen) to yield effector plasmids. The transient expression assay was performed with Arabidopsis protoplasts as previously described (Asai *et al.*, 2002). For each transformation, 5 µg of reporter plasmid and 4 µg of effector plasmid were used. For normalization of the activity of the reporter gene, 0.5 µg of plasmid pRLC (Wang *et al.*, 2010) was used as the internal control.

### Sequence alignment and phylogenetic analysis

Amino acid sequences of HDL, ARRs, and homologs were aligned using Clustal W, and a Neighbor–Joining phylogenetic tree was constructed using MEGA 4 software. The most parsimonious trees with bootstrap values from 1000 trials are shown.

### Accession numbers or gene identifiers used in this study

Sequence data used in this study were retrieved from GenBank and can be found under the following accession numbers: *HDL* (Medtr5g021930), *MtTPL* (Medtr4g009840), *STF* (Medtr8g107210), *LFL* (Medtr7g060630), *WUS* (At2g17950), *WOX1* (At3g18010), *WOX2* (At5g59340), *WOX3/PRS* (At2g28610), *WOX4* (At1g46480), *WOX5* (At3g11260), *WOX6/PFS* (At2g01500), *WOX7* (At5g05770), *WOX8* (At5g45980), *WOX9* (At2g33880), *WOX10* (At1g20710), *WOX11* (At3g03660), *WOX12* (At5g17810), *WOX13* (At4g35550), *WOX14* (At1g20700), *ARR3* (At1g59940), *ARR4* (At1g10470), *ARR5* (At3g48100), *ARR6* (At5g62920), *ARR7* (At1g19050), *ARR9* (At3g57040), *ARR15* (At1g74890), *ARR16* (At2g40670), *ARR17* (At3g56380), and *ARR22* (At3g04280).

## Results

### Identification and characterization of the *M. truncatula headless* mutant

**Fig. 1.**
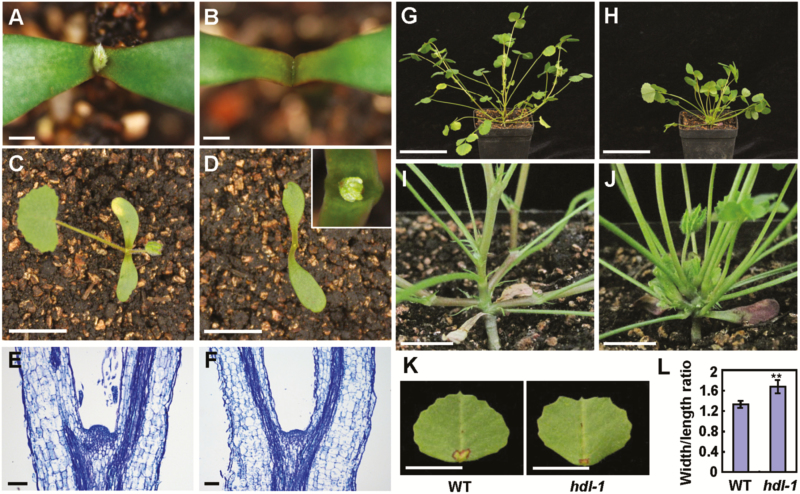
Morphological comparison between the wild type and *hdl* plants. (A, B) Phenotype of the wild type (A) and *hdl-1* mutant (B) at 3 days after germination (DAG). Scale bars=1 mm. (C, D) Phenotype comparison of the wild type (C) and *hdl-1* mutant (D) at 8 DAG. The inset shows magnification of the shoot apex in *hdl-1*. Scale bars=1 cm. (E, F) Longitudinal sections of the wild type (E) and *hdl-1* mutant (F) shoot apices at 3 DAG. Scale bars=100 µm. (G, H) Five-week-old wild-type (G) and *hdl-1* mutant (H) plants. Scale bars=5 cm. (I, J) Close-up views of the basal part of wild-type (I) and *hdl-1* mutant (J) plants in (G, H). Scale bars=1 cm. (K) Dissected leaves of the wild type and *hdl-1* mutant. Scale bars=1 cm. (L) Comparison of leaf width/length ratio in the wild type and *hdl-1* mutant. Bars represent means ±SE (*n*=30 plants). The asterisks indicate significant differences (** *P*<0.01, Student *t*-test).

**Fig. 2.**
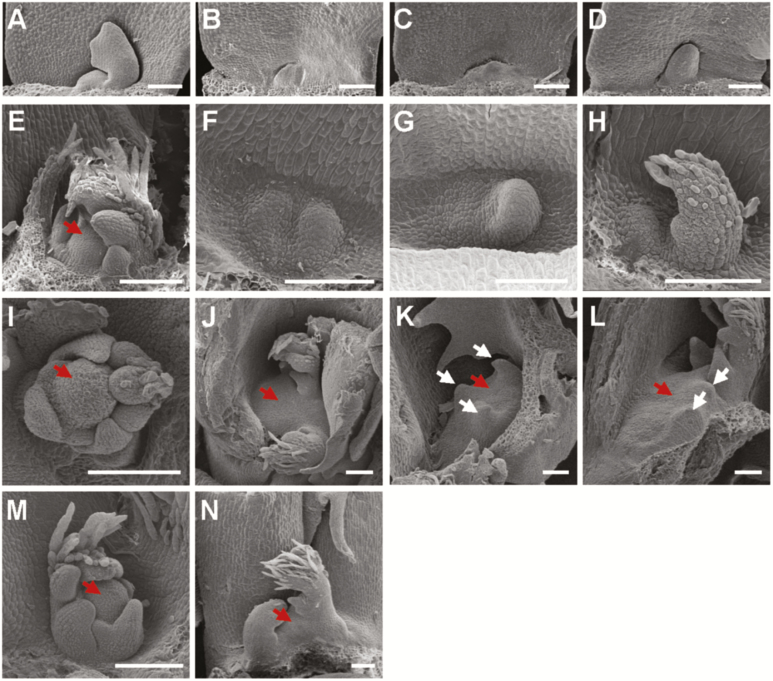
Shoot meristem phenotypes of the *hdl-1* mutant. (A–D) Scanning electron micrographs of the wild-type (A) and *hdl-1* apices after germination for 12 h (B–D). Scale bars=100 µm. (E–H) Shoot apices of 4-day-old wild type (E) and *hdl-1* (F–H). The red arrow points to the shoot apical meristem. Scale bars=100 µm. (I–L) Shoot apices from 35-day-old wild-type (I) and *hdl-1* (J–L) plants. Red arrows point to the shoot apical meristem, and white arrows indicate leaf primordia. Scale bars=100 µm. (M, N) Axillary buds from 35-day-old wild-type (M) and *hdl-1* (N) plants. Red arrows point to the axillary meristem. Scale bars=100 µm.

To understand the molecular mechanisms that determine the development of the SAM in legumes, three identical SAM-defective mutants named *headless* (*hdl-1*, *hdl-2*, and *hdl-3*) with stemless and bushy phenotypes were identified from forward genetic screens of *Tnt1* retrotransposon-tagged lines of *M. truncatula* genotype R108 (Tadege *et al.*, 2015). The *hdl* mutant never bolts in at least 2 years and continuously produces only leaves. In contrast, the wild-type R108 produces the first metamer within the first week of growth after germination, and takes ~6 weeks to form the first flowers. The *hdl* mutant plants show a range of obvious defects in meristem maintenance and leaf formation at the early seedling stage, which become apparent at the simple (unifoliate) leaf stage. *Medicago truncatula* produces trifoliate leaves but the first true leaf, which probably has an embryonic origin, is always simple. In *hdl* mutants, the unifoliate leaf is either absent or significantly delayed in appearance compared with wild-type R108 (Fig. 1A–D; Supplementary Figs S1, S2A, B). Histological analysis showed that *hdl* mutants initiate a defective SAM that is essentially flat at the early stage of seedling development, leading to retarded growth compared with the wild type (Figs 1E, F, 2A–J). At a later stage, several leaf primordia are initiated ectopically from the abnormal shoot meristems on the flat apex (Fig. 2K, L). Gradually, more and more leaves start to form from the axil of cotyledons (Supplementary Fig. S2A–D) and axils of mutant leaves, but there is no apparent stem elongation (Fig. 1G–J). Moreover, dome-shaped structures (presumably ectopic meristems) are formed at the petiole base of older *hdl* leaves, and multiple leaves are initiated from these apical domes in a disorganized manner (Supplementary Fig. S2E). In addition, *hdl* mutants also show defects in axillary mersitem development which usually produce up to three leaves before the meristematic activity terminates (Fig. 2M, N). Eventually the *hdl* mutants remain bushy and dwarf, being locked at the vegetative phase without any bolting or stem elongation throughout their life, appearing forever young at least for the 2 years that we tested, suggesting severe compromise in SAM organization and maintenance. Both the Arabidopsis *wus-1* and Petunia *terminator* (*ter*) mutants exhibit premature termination of the vegetative and floral meristems but display reiteration of ectopic meristems and leaves in a ‘stop-and-go’ type of growth with stems and inflorescences (Laux *et al.*, 1996; Stuurman *et al.*, 2002). In addition, *hdl* mutant leaves lack leaf elongation in the longitudinal direction, leading to a reduced leaf tip and a heart-shaped blade morphology with increased width/length ratio compared with R108 (Fig. 1K, L). Occasionally, the *hdl* mutant leaves show defects in the initiation of more than three leaflets (Supplementary Fig. S3). These leaf phenotypes indicate that *HDL* may function in the determinate leaf primordia. These novel and conserved phenotypes of *hdl* mutants show that *HDL* function is highly relevant to SAM maintenance and regulation of leaf blade development in *M. truncatula*.

### Molecular cloning and expression pattern of the *HDL* gene

**Fig. 3.**
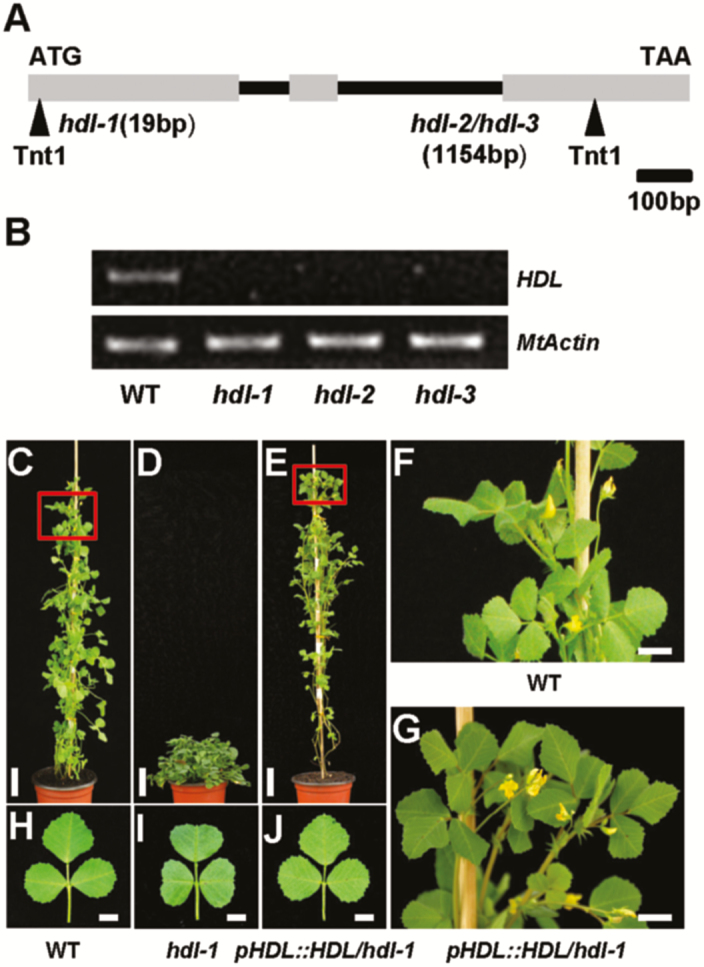
Molecular cloning of the *HDL* gene. (A) Schematic representation of the gene structure of *HDL* showing the *Tnt1* insertion sites in *hdl* mutants. (B) RT–PCR analysis of *HDL* transcripts in the wild-type (WT) and *hdl* alleles. (C–E) Phenotypes of wild-type (C), *hdl-1* mutant (D), and T_1_ transgenic seedlings of *hdl-1* complemented with *pHDL::HDL* (E). Scale bars=5 cm. (F, G) Magnification of the indicated region in (C, E), respectively. Scale bar=1 cm. (H–J) Dissected leaves of wild-type (H), *hdl-1* mutant (I), and T_1_ transgenic seedlings of *hdl-1* complemented with *pHDL::HDL* (J). Scale bars=0.5 cm.

We cloned the *HDL* gene by PCR-based genotyping of flanking sequence tags (FSTs) in segregating populations. Flanking sequence analysis of *Tnt1* retrotransposon in the *hdl-1* mutant revealed that FST 17 segregated with the mutant phenotype. The *hdl-1* mutant phenotype is determined by a single recessive gene, and all mutant plants genotyped homozygous for FST 17 show the phenotype representing the *hdl* locus. The genomic sequence of FST 17 (*HDL*) contains three exons, and the *Tnt1* retrotransposon is inserted at the beginning of exon 1, 19 bp downstream of the translational start in the *hdl-1* mutant (Fig. 3A). We further confirmed by PCR analysis that the other two identical phenotype mutants, *hdl-2* and *hdl-3*, are allelic to *hdl-1*, and found that both have *Tnt1* insertions at the same position in exon 3 of *HDL*, 1151 bp downstream of the translational start, even though *hdl-2* and *hdl-3* were identified from independent *Tnt1*-tagged lines (Fig. 3A). RT–PCR analysis of the transcript in mutant seedlings revealed that the transcription of the full-length coding sequence of *HDL* is abolished in all three *hdl* mutants, while expression was clearly detected in the R108 control (Fig. 3B). To confirm further that the *hdl* mutant phenotype is caused by the disruption of this locus, we crossed heterozygous *hdl-1* and *hdl-2* plants and generated *hdl-1*/*+ hdl-2*/*+* F_1_ progeny, which displayed identical stemless phenotypes to *hdl-1* and *hdl-2* (Supplementary Fig. S4), indicating that *hdl-2* and *hdl-3* are allelic to *hdl-1*. The identity of *HDL* was further confirmed by a mutant complementation test. The plasmid *pHDL::HDL gDNA* containing a 5.4 kb genomic DNA fragment consisting of a 2713 bp upstream sequence, the entire 1346 bp *HDL* gene including three exons and two introns, and a 1366 bp downstream region was introduced into the *hdl-1* mutant. We also transformed the *hdl-1* line with the *p35S::HDL* (CDS) construct. Phenotypic examination of transgenic plants showed that the *hdl-1* mutant is complemented by the *pHDL::HDL* construct (Fig. 3C–J), while it is poorly rescued by the *p35S::HDL* construct. The *p35S::HDL* transgenic plants show partial recovery of bolting, but the leaf shape is even more poorly restored compared with the *hdl* mutant (Supplementary Fig. S5), suggesting that HDL function is orchestrated by its specific spatiotemporal expression pattern.

**Fig. 4.**
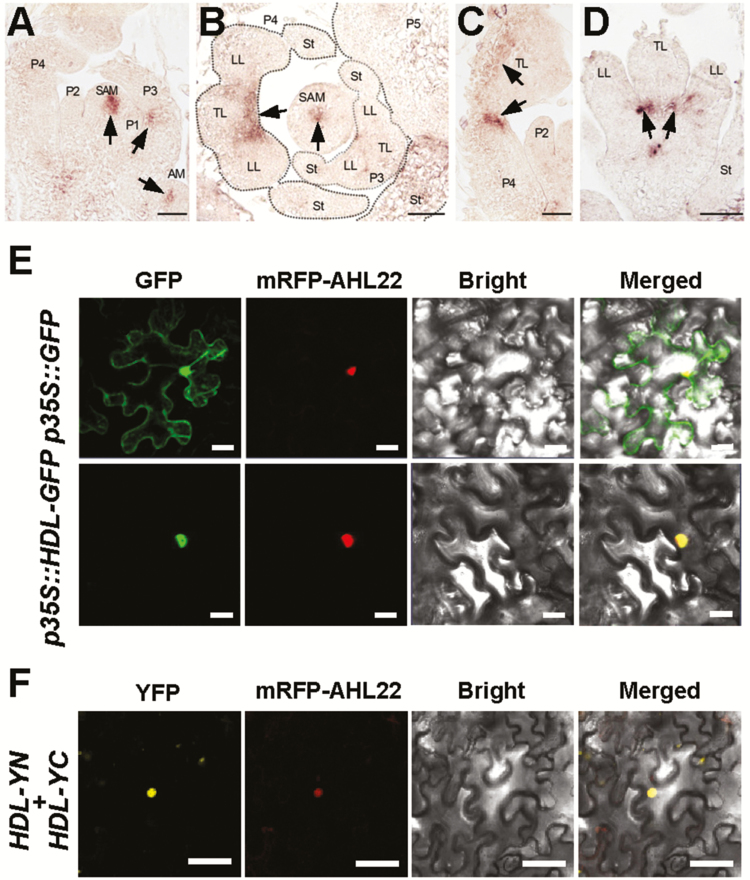
Expression pattern of *HDL* and subcellular localization of the HDL protein. (A) *HDL* expression by mRNA *in situ* hybridization in 4-week-old vegetative shoot apex viewed in longitudinal sections. SAM, shoot apical meristem; AM, axillary meristem. P1–P4 indicate different leaf primordia stages. Arrowheads point to signals. Scale bar=50 μm. (B) *In situ* hybridization of *HDL* in the cross-section of the vegetative shoot apex. SAM, shoot apical meristem; TL, terminal leaflet; LL, lateral leaflet; St, stipule. P3–P5 indicate different leaf primordia stages. Arrowheads point to signals. Scale bar=50 μm. (C) *In situ* hybridization of *HDL* in the longitudinal section of P4 leaf primordia. TL, terminal leaflet. P2 and P4 indicate different leaf primordia stages. Arrowheads point to signals. Scale bar=50 μm. (D) *In situ* hybridization of *HDL* in the cross-section of P4 leaf primordia. TL, terminal leaflet; LL, lateral leaflet; St, stipule. Arrowheads point to signals. Scale bar=50 μm. (E) Subcellular localization of *35S::GFP* and *35S::HDL-GFP* in tobacco epidermal cells. Nuclear protein AHL22 was used as a nuclear localization marker. Scale bars=50 µm. (F) BiFC assay showing that HDL could form a homodimer. YN indicates the N-terminal half of YFP and YC indicates the C-terminal half of YFP. Nuclear protein AHL22 was used as a nuclear localization marker. Scale bars=50 μm.

Sequence alignment and phylogenetic analysis revealed that *HDL* encodes a 302 amino acid WOX family homeodomain transcriptional regulator with 40% amino acid identity to Arabidopsis WUS (Supplementary Fig. S6). The predicted HDL protein contains the highly conserved homeodomain near the N-terminus, an identical WUS-box, as well as an EAR-like motif and acidic region at the C-terminal region (Supplementary Fig. S7) similar to WUS. RNA *in situ* hybridization in the vegetative shoot apex of wild-type R108 revealed that *HDL* is specifically expressed in the central region of the SAM and the axillary meristem (Fig. 4A, B; Supplementary Fig. S8), which is consistent with previous reports (Chen *et al.*, 2009; Kurdyukov *et al.*, 2014). Interestingly, *HDL* expression is also detected in the central/joint region of leaf primordia at the P3 stage (Fig. 4A). In the older primordia stage P4, *HDL* is expressed in the proximal–distal axis of the leaf blade (Fig. 4C) and at the joint region of the leaflets (Fig. 4C, D). These specific expression patterns of *HDL* are consistent with its function in regulating SAM maintenance and leaf blade development during *M. truncatula* shoot morphogenesis.

### *HDL* encodes a nuclear-localized transcriptional repressor

To determine the subcellular localization of the HDL protein, we fused the C-terminus of HDL with green fluorescent protein (GFP) under the control of the *Cauliflower mosaic virus* (CaMV) 35S promoter, and the construct was transferred into tobacco (*N. benthamiana*) leaf epidermal cells by the *Agrobacterium* infiltration method. In contrast to the GFP control, which is localized to both the cytoplasm and nucleus of epidermal cells, the HDL–GFP fusion protein was localized in the nucleus (Fig. 4E). In addition, we found that HDL could form a homodimer in the nucleus through BiFC analysis using split yellow fluorescent protein (YFP) (Fig. 4F), which is consistent with previous reports that WUS homodimerization is possibly important for its regulation of stem cell activity (Daum *et al.*, 2014).

**Fig. 5.**
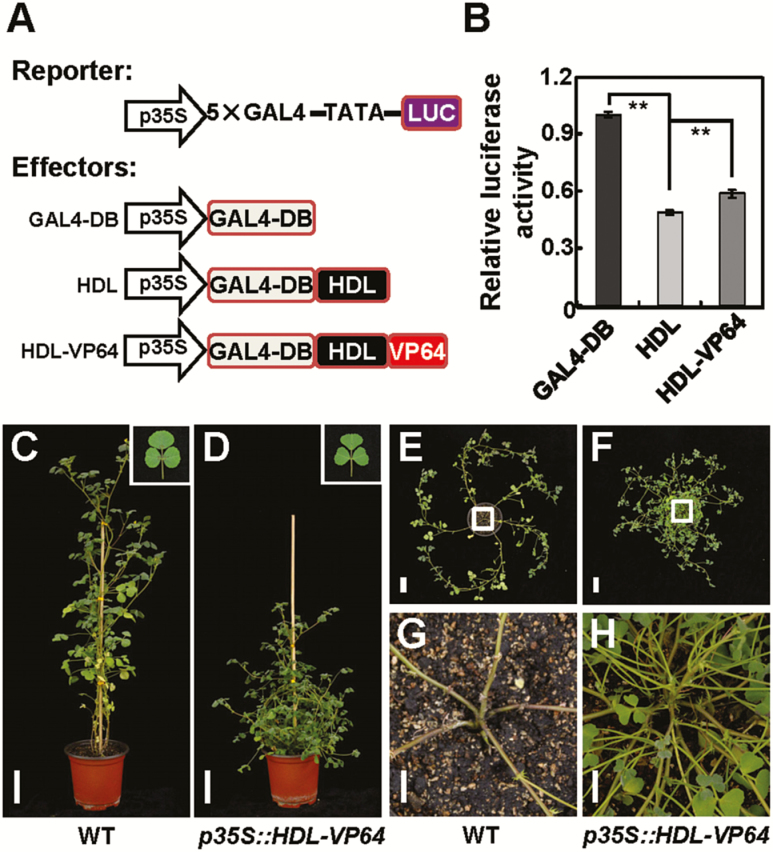
HDL exhibits transcriptional repressive activity. (A) Schematic representation of reporter and effector constructs used in the transient expression assay. (B) Relative luciferase activities measured as bioluminescence in Arabidopsis protoplasts. Error bars represent the SE of three replicate assays. The asterisks indicate significant differences (**P<0.01, Student *t*-test). (C–H) Phenotypes of wild-type (C, E, G) and *p35S::HDL-VP64* transgenic plants (D, F, H). (G) and (H) are magnifications of the indicated regions in (E) and (F), respectively. Scale bars=5 cm in (C–F), 1 cm in (G, H).

To investigate whether HDL functions as a transcriptional activator or repressor, we examined its transcriptional activity by dual luciferase transient expression assay in Arabidopsis protoplasts. The effector plasmid was constructed by fusing the GAL4 DNA-binding domain to the N-terminus of HDL, and the reporter plasmid contained the luciferase (*LUC*) gene fused to a 5×GAL4-binding site (Fig. 5A). Bioluminescence measurements showed that luciferase activity is reduced by ~2-fold in the presence of HDL effector protein (Fig. 5B), indicating its strong repressive activity. These results are consistent with previous findings that Arabidopsis *WUS* encodes a nuclear-localized transcriptional repressor (Kieffer *et al.*, 2006).

To confirm the repressive function of HDL in the regulation of SAM maintenance and leaf blade development, we fused the exogenous activation domain VP64 to the C-terminus of HDL, which reduces its repressive activity (Fig. 5A, B), under the control of the 35S promoter and transformed this construct into *M. truncatula* ecotype R108. We found that 16 of 21 *p35S::HDL-VP64* transgenic plants exhibited reduced apical dominance as well as heart-shaped blade morphology (Fig. 5C–H), which partially mimic phenotypes of the *hdl* mutant, indicating a dominant negative effect of the chimeric HDL–VP64 in SAM and leaf development. This is not caused by co-suppression as the endogenous *HDL* transcript level is not altered (Supplementary Fig. S9), but by the antagonistic activation activity of VP64. Taken together, these results indicate that HDL may mainly act as a transcriptional repressor in regulating *M. truncatula* SAM maintenance and leaf development.

### HDL physically interacts with the co-repressor MtTPL protein using its conserved WUS-box and EAR-like motif

**Fig. 6.**
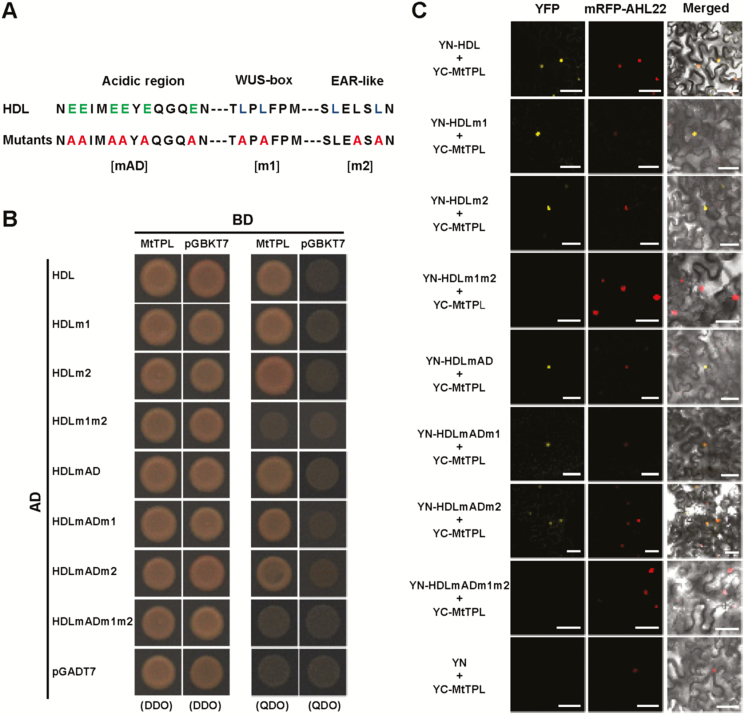
HDL physically interacts with the co-repressor MtTPL through its conserved WUS-box and EAR-like motif. (A) Sequence of the acidic region, WUS-box, and C-terminal EAR-like motif in the HDL protein. Mutations introduced into the acidic region (mAD), WUS-box (m1), and EAR-like motif (m2) are indicated by red fonts. (B, C) Identification of HDL domains required for interaction with MtTPL by the yeast two-hybrid system and BiFC assay. DDO indicates SD/-Leu/-Trp; QDO indicates SD/-Trp/-Leu/-His/-Ade; YN indicates the N-terminal half of YFP, and YC indicates the C-terminal half of YFP. Nuclear protein AHL22 was used as a nuclear localization marker. Scale bars=50 µm.

Since there is a conserved WUS-box and EAR-like motif in the C-terminus of HDL (Fig. 6A; Supplementary Fig. S7), we examined the interaction between HDL and the co-repressor MtTPL by performing Y2H and BiFC assays (Fig. 6B, C). We found that HDL interacts with MtTPL in the nucleus reconstituting the yellow fluorescence when both HDL-YN and MtTPL-YC proteins are transiently expressed in *N. benthamiana* leaf epidermal cells (Fig. 6C). This interaction between HDL and MtTPL in split YFP is consistent with the Y2H analysis in quadruple drop-out medium (QDO) (Fig. 6B). Moreover, we found that either substitution of acidic amino acids in the acidic region (mAD) or mutation of leucine residues in the WUS-box and the EAR-like motif alone does not obviously reduce its interaction with MtTPL, but combined mutations in both the WUS-box and EAR-like motif abolish the interaction with Mt-TPL in Y2H assay (Fig. 6B). This finding was further verified by BiFC assay, in which the HDLm1m2-YN coupled with MtTPL-YC fails to generate YFP signals despite both proteins being stably expressed in tobacco cells (Fig. 6C; Supplementary Fig. S10), demonstrating that both the WUS-box and EAR-like motif are required for the HDL interaction with MtTPL.

### Transcript abundance of A-type cytokinin-responsive genes in shoot apices of the *hdl* mutant

**Fig. 7.**
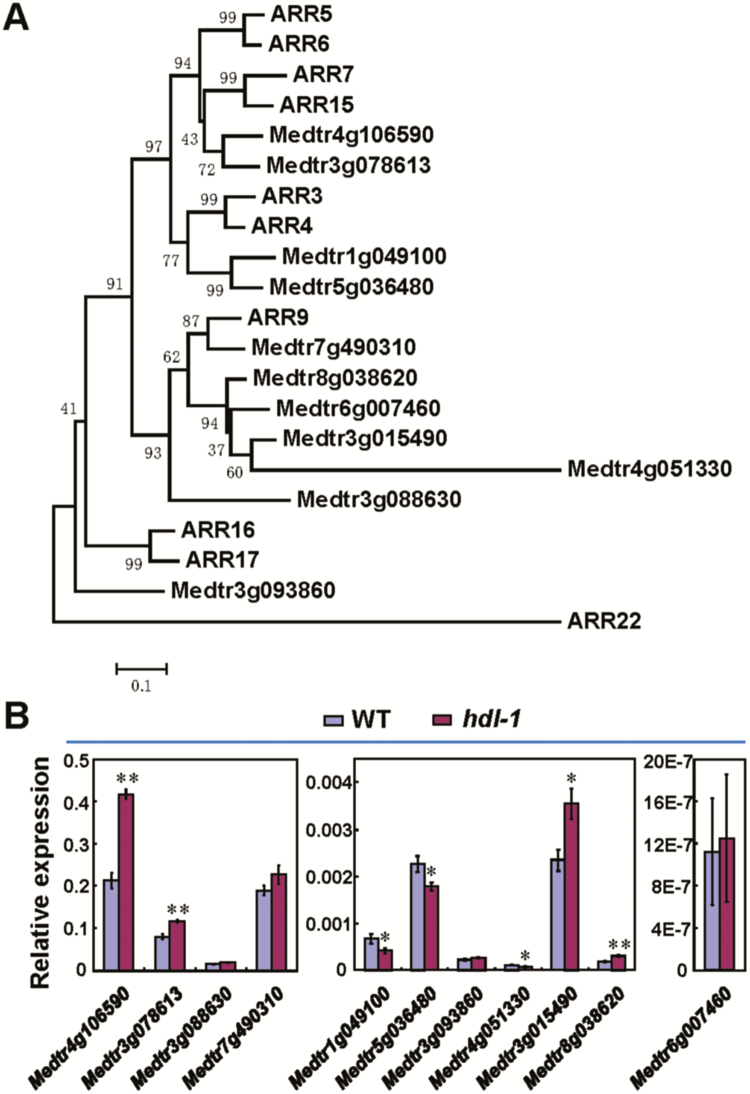
Transcript abundance of A-type cytokinin-responsive genes in the *hdl-1* mutant. (A) Phylogenetic analysis of A-type cytokinin-responsive regulators in Arabidopsis and *M. truncatula*. (B) Transcript levels of A-type cytokinin-responsive genes in shoot apices of the *hdl-1* mutant. Error bars represent the SE of three replicate experiments. The asterisks indicate significant differences (*P<0.05, **P<0.01, Student *t*-test) compared with the wild type.

The *ARR* genes have been reported to be direct targets of WUS, and the transcript of several A-type cytokinin-responsive genes including *ARR5*, *ARR6*, *ARR7*, and *ARR15* is up-regulated in the Arabidopsis *wus* mutant (Leibfried *et al.*, 2005). Because HDL functions similarly to WUS in regulating shoot meristem development, we wondered whether this would be the case in *M. truncatula* as well. A total of 11 putative A-type cytokinin response regulators were isolated using the Arabidopsis A-type ARR proteins as a BLAST query in the National Center for Biotechnology Information (NCBI) and J. Craig Venter Institute (JCVI) (Fig. 7A). We found that the expression levels of 4 out of 11 type-A ARR family members are considerably increased in *hdl-1* shoot apices (Fig.7B). Among them, from the phylogenic analysis (Fig. 7A), it was found that Medtr4g106590 and Medtr3g078613 are close to ARR7 and ARR15, while Medtr3g015490 and Medtr8g038620 group together with ARR9. The up-regulation of ARR7 and ARR15 homologs in *hdl* is consistent with the finding in the Arabidopsis *wus-1* mutant (Leibfried *et al.*, 2005) and suggests that HDL may modulate the cytokinin response for its function in meristem maintenance. We also found that 3 out of 11 type-A ARR family members, Medtr1g049100, Medtr5g036480, and Medtr4g051330, are slightly down-regulated in *hdl-1* (Fig. 7B), suggesting that *HDL* has a complex relationship with these cytokinin signaling response regulators.

### Genetic analysis of *HDL* and *STF* in *M. truncatula* leaf development

**Fig. 8.**
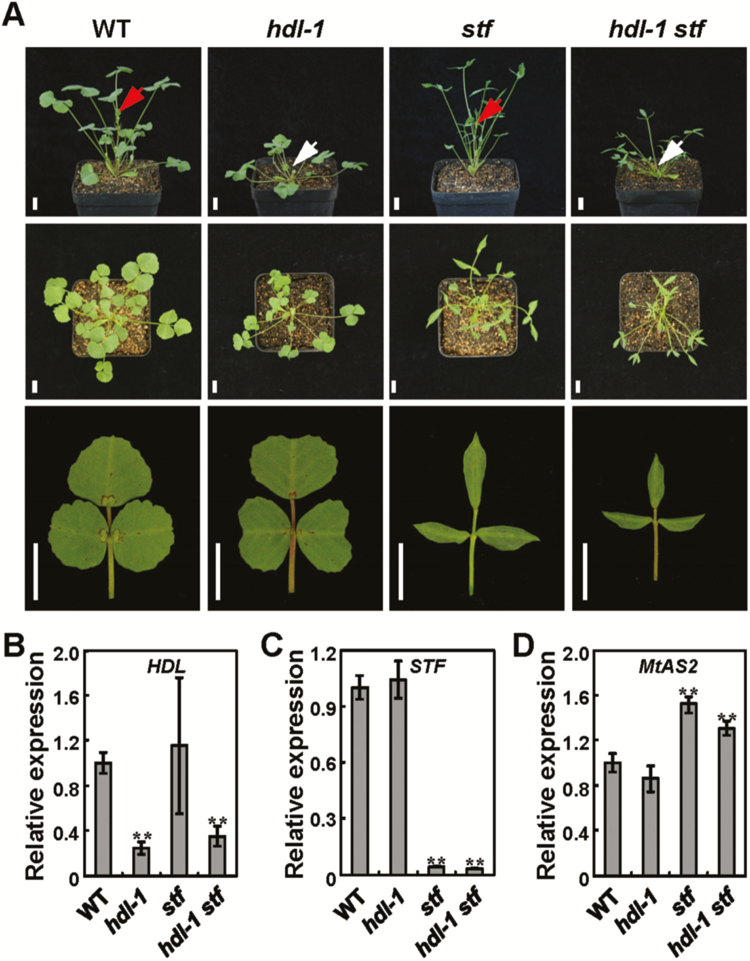
Genetic analysis of *HDL* and *STF* in regulating leaf development. (A) Phenotype analysis of the wild type, *hdl-1*, *stf*, and the *hdl-1 stf* double mutant. Scale bars=1 cm. (B–D) Transcript levels of *HDL* (B), *STF* (C), and *MtAS2* (D) in young leaves of the wild type, *hdl-1*, *stf*, and the *hdl-1 stf* double mutant. Error bars represent the SE of three replicate experiments. The asterisks indicate significant differences (*P<0.05, Student *t*-test) compared with the wild type.

We previously reported that the *M. truncatula* WOX gene *STF* mainly functions as a transcriptional repressor in regulating leaf blade outgrowth (Tadege *et al.*, 2011; Lin *et al.*, 2013*a*). The obvious leaf phenotype of *hdl* and the fact that both HDL and STF act as transcriptional repressors by interacting with the co-repressor MtTPL prompted us to test the genetic relationship between *HDL* and *STF* in regulating leaf development in *M. truncatula*. We generated the *hdl-1 stf* double mutant using heterozygotes and compared the phenotypes of single and double mutants *hdl-1*, *stf*, and *hdl-1 stf*. Compared with the single mutants, the *hdl-1 stf* double mutant shows an additive effect combining the *hdl* stemless phenotype and the *stf* narrow leaf phenotype (Fig. 8A). In the *hdl* mutant, the elongation of the leaf tip is reduced, leading to a heart-shaped leaf with an increased width/length ratio. In the *stf* mutant, the leaf length elongation is virtually unaffected and the distal leaf tip is characteristically pointed due to absence of lateral expansion, but in the *hdl-1 stf* double mutant, leaves show a combination of the narrow blade, distorted margin, and pointed tip of *stf*, and the reduced blade length of *hdl* (Fig. 8A), suggesting that HDL and STF may function in independent genetic pathways in leaf development. In agreement with this genetic analysis, quantitative RT–PCR analysis showed that the expression level of *STF* is not significantly changed in the young leaves of *hdl* compared with the wild type, and vice versa (Fig. 8B, C). As *STF* regulates leaf lateral extension by partially repressing the expression of the adaxial polarity factor *ASYMMETRIC LEAVES2* (*AS2*) through its interaction with MtTPL in *M. truncatula*, the expression of *MtAS2* was further compared in *hdl-1*, *stf*, and *hdl-1 stf*. Quantitative RT–PCR results showed that there is no obvious change of *MtAS2* expression in *hdl-1* young leaves compared with the wild type, and expression of *MtAS2* in the *hdl-1 stf* double mutant is similar to that in *stf* (Fig. 8D). These results suggest that HDL and STF have distinct functions in *M. truncatula* leaf blade development.

## Discussion

*WUSCHEL* is the founding member of the WOX family and plays an essential role in maintaining stem cell identity in the SAM of plants (Laux *et al.*, 1996; Mayer *et al.*, 1998; van der Graaff *et al.*, 2009). Although this key stem cell regulator has been isolated and to some extent characterized from several plant species, its actual function is described by a genetic mutation only in a couple of cases apart from Arabidopsis, which are not necessarily telling the same story. In this study, we described an *M. truncatula* stemless and bushy mutant named *headless* (*hdl*), in which shoot meristem maintenance is disrupted by *Tnt1* retrotransposon insertion in the exons of the *HDL* gene. *HDL* encodes a nuclear-localized homeodomain transcription factor closely related to Arabidopsis WUS. Loss of *HDL* function led to arrested and disorganized shoot meristem development including the SAM and any axillary meristems, resulting in a stemless phenotype (Fig. 1G–J). The only visible above-ground structures in the mutant are leaves and stipules at all stages of development, but the leaves also show a characteristic heart shape compared with the more oval-shaped blade in the wild-type R108, unlike any of the reported *wus-1*, *ter*, and *roa* mutants (Laux *et al.*, 1996; Stuurman *et al.*, 2002; Kieffer *et al.*, 2006).

Wild-type *M. truncatula* shows a trailing type of growth (Bucciarelli *et al.*, 2006) in which the SAM first produces a single unifoliate leaf after germination, followed by establishment of a central growth axis. The central growth axis then produces four trifoliate leaves in close succession and at the axils of each leaf is an axillary meristem (the fourth leaf of which is the SAM), which develop into growing units called metamers (main stems), each with elongating internodes, trifoliate leaves, and axillary buds (meristems). These axillary meristems have the potential to grow into secondary metamers and may branch out until terminated later with the development of the inflorescence meristem, giving rise to a prostrate habit. The *hdl* mutant partially fails in the formation of the unifoliate leaf as some of the mutant plants skip this first leaf altogether, and completely fails in the proper establishment of the central growth axis. The *hdl* SAM appears essentially flat 3 d after germination, while the wild type has a clear apical dome (Fig. 1E, F). Gradually, a disorganized SAM starts to form in *hdl* with fewer cells, producing fewer trifoliate leaves (two or three) around it, but perhaps all the meristematic cells are consumed in making the leaves, and the SAM is unable to replenish itself to maintain a central axis with a rib meristem. However, unorganized ectopic meristems are formed at the leaf axils, reminiscent of axillary meristems in the wild type, but again these end up forming only leaves instead of metamers, and the cycle continues with indefinite proliferation of leaves, giving the *hdl* mutant a bushy and ‘forever young’ appearance. These results indicate that *HDL* is a key regulator required for organization and maintenance of the SAM and axillary meristems in *M. truncatula.* Recent studies also showed that Arabidopsis *WUS* is not only necessary for SAM maintenance but is also required for AM initiation. Nevertheless, the *hdl* meristem phenotypes appear to be stronger than the Arabidopsis *wus-1* and the equivalent Petunia *ter* phenotypes. Both the *wus-1* and *ter* SAMs are correctly specified at the beginning, and mutants are indistinguishable at the early seedling stage from their corresponding wild-type seedlings, but the meristem terminates prematurely (Laux *et al.*, 1996; Stuurman *et al.*, 2002), and that is where the phenotypes become obvious. Not only that, but the SAM re-establishes itself and resumes growth in both cases, producing vegetative and inflorescence stems in a ‘stop-and-go’ type of growth (Laux *et al.*, 1996; Stuurman *et al.*, 2002). In addition, ectopic meristems in *wus-1* mutants form aerial rosettes and inflorescences, reiterating growth (Laux *et al.*, 1996). For this reason, WUS is thought to be neither necessary nor sufficient for stem cell specification (Green *et al.*, 2005). Our results highlight that *M. truncatula HDL* is necessary for both correct specification and maintenance of the SAM and axillary meristems since all shoot meristems are completely abrogated in the *hdl* mutant, uncovering novel insights in meristem biology. In rice, the ortholog of *WUS*, *MONOCULM 3* (*MOC3*)/*TILLERS ABSENT1* (*TAB1*), has been shown to be required for the maintenance of the pre-meristem zone and the formation of axillary meristem instead of the SAM (Lu *et al.*, 2015; Tanaka *et al.*, 2015). Disruption of *MOC3*/*TAB1* leads to axillary meristem arrest, resulting in a monoculm phenotype with a normal culm (primary stem) (Tanaka *et al.*, 2015), suggesting diversification of *WUS* functions in regulating the stem cell niche in monocot species.

In Arabidopsis, the WUS protein acts mainly as a repressor in stem cell regulation, and the WUS-box is the essential repressive domain (Ikeda *et al.*, 2009). Sequence alignment reveals that the HDL protein contains a conserved WUS-box and EAR-like motif (Fig. 6A; Supplementary Fig. S7). We found that HDL exhibits a strong repressive activity and physically interacts with the transcriptional co-repressor MtTPL using the conserved WUS-box and EAR-like motif (Figs 5B, 6B, C), suggesting that HDL is also primarily a transcriptional repressor. This is consistent with the proposal that all WUS/modern clade WOX members with the conserved WUS-box may primarily function as transcriptional repressors through forming a WOX–TPL repressor complex shown for WOX1 and WOX3 homologs, STF and LFL (Lin *et al.*, 2013a, b; Zhang *et al.*, 2014; Niu *et al.*, 2015). This WOX–TPL interaction was also recently reported to be important for WOX5 function in the Arabidopsis root apical meristem, suggesting a role in columella stem cell maintenance (Pi *et al.*, 2015). Moreover, we found that the HDL protein could form a homodimer, which is consistent with the previous report that the formation of WUS dimers might contribute to the regulation of shoot apical stem cell activity (Daum *et al.*, 2014).

The phytohormone cytokinin plays an essential role in regulating plant developmental programs including meristem function. A previous report showed that disruption of the *LONELY GUY* (*LOG*) gene, which encodes a cytokinin-activating enzyme, causes the arrest of SAM activity in rice (Kurakawa *et al.*, 2007). In maize, mutation of the cytokinin-inducible gene *ABPHYL1* (*ABPH1*) leads to increased meristem size. ABPH1 is homologous to two-component response regulators and is proposed to play a role in negative regulation of cytokinin response (Giulini *et al.*, 2004). In the Arabidopsis shoot meristem, *KNOX* and *WUS* promote meristem activity in part through activating cytokinin signaling (Jasinski *et al.*, 2005; Shani *et al.*, 2006). Cytokinin signaling activates *WUS* transcription (Zhang *et al.*, 2017; H. Wang *et al.*, 2017), and *WUS* in turn activates direct repression of type-A ARR negative regulators of cytokinin signaling (Leibfried *et al.*, 2005). Our results showed that four genes related to *ARR7*, *ARR9*, and *ARR15* are significantly up-regulated in the *hdl* mutant shoot apex (Fig. 7A, B), suggesting that *HDL* is required for repressing type-A *ARR* genes in *M. truncatula* shoot meristems. It is likely that *WUS* and *HDL* share this conserved mechanism to modulate cytokinin signaling for stem cell maintenance in their respective SAMs. However, we also noted that other type-A response regulators including *Medtr1g049100*, *Medtr5g036480*, and *Medtr4g051330*, related to *ARR3*, *ARR4*, and *ARR9*, respectively, are down-regulated (Fig. 7A, B), necessitating further characterization of these genes in *M. truncatula*. Whether species-specific sensitivity variation in the cytokinin signal or the diversity of cytokinin response regulators and their effect in modulating the cytokinin signal, or others factors unrelated to cytokinin signaling, contribute to the complete arrest of the SAM and axillary meristems in *hdl* mutants compared with *wus-1* mutants is unknown at this stage. The mechanism by which *HDL* regulates leaf shape in *M. truncatula* also remains to be elucidated.

It has been reported that *WOX1*, *WOX3*, and their orthologs are major regulators in controlling lateral leaf blade outgrowth in diverse eudicot and monocot plants (Scanlon *et al.*, 1996; Nardmann *et al.*, 2004; Vandenbussche *et al.*, 2009; Tadege *et al.*, 2011; Nakata *et al.*, 2012; Zhuang *et al.*, 2012; Cho *et al.*, 2013; Ishiwata *et al.*, 2013). Although the primary function of *WUS* is regulating the maintenance of vegetative and inflorescence meristems (Laux *et al.*, 1996; Mayer *et al.*, 1998), it is also involved in ovule development and floral patterning in the differentiated floral organs (Lohmann *et al.*, 2001). Other clues also implicate that the founding member of the WOX family may be involved in leaf blade development. We previously showed that the leaf blade phenotypes of *stf* in *M. truncatula* and *lam1* in *N. sylvestris* could be fully restored by expressing Arabidopsis *WUS* under the control of the *STF* promoter (Tadege *et al.*, 2011; Lin *et al.*, 2013*a*), suggesting that *WUS* can function in determinate leaf primordia, and the leaf and shoot apical meristems might share a common mechanism. It is even likely that WUS has a redundant function in leaf blade development with other *WOX* genes. The *wox1 prs wus* triple mutant exhibits a noticeably narrower leaf blade compared with the *wox1 prs* double mutant, implicating that *WUS* might function in regulating leaf lateral blade outgrowth in Arabidopsis (Zhang and Tadege, 2015), although this has not been investigated in detail. However, in *M. truncatula*, the mutation of *HDL* results in clearly altered leaf shape, changing from a more or less oval shape in R108 to heart-shaped blades in the mutant, suggesting that *HDL* may regulate leaf development in the proximal–distal axis in contrast to the role of *STF* in regulating blade outgrowth in the medial–lateral axis. We found that the *stf hdl* double mutant leaf phenotypes are additive (Fig. 8), suggesting independent genetic pathways for leaf width and length growth in *M. truncatula*. It is possible that the *WUS/HDL* function in stem cell maintenance or lateral organ patterning may depend on dosage, targets, and the microenvironment. Identification of downstream targets of *HDL* responsible for leaf development will shed light on understanding the underlying molecular mechanism of HDL function in leaf length regulation and any commonalities that exist with its major function in meristem maintenance.

A potential problem in the recruitment of leaf primordia founder initial cells was seen at the early seedling stage where establishment of the unifoliate leaf is significantly delayed or sometimes blocked. However, once the first true leaves are formed, this problem appears to dissipate and the *hdl* mutant leaves maintain their trifoliate identity, suggesting that the *hdl* leaf shape phenotype is unlikely to be caused by defects in the recruitment of leaf primordia founder initials from the SAM. Occasionally, leaves may acquire extra leaflets but, in most cases, trifoliate leaves are continuously formed from the *hdl* SAM and axillary meristems. However, in doing so, the stem cells are fully consumed and unable to replenish themselves. Similar to *WUS*, *HDL* may not be required for SAM initiation (Zhang *et al.*, 2017) but, unlike the *wus-1* mutant, normal SAM function is never observed in the extended life of the *hdl* mutant, suggesting that the central zone and the rib zone of the SAM are probably defective from the outset. These observations suggest that *M. truncatula HDL* is required for both shoot meristem organization and stem cell maintenance, as well as for leaf elongation in the length direction but not for allocation of leaf founder cells from the stem cell pool, uncovering novel aspects of meristem biology.

## Supplementary data

Supplementary data are available at *JXB* online.

Fig. S1. The *hdl* mutant shows a defect in leaf outgrowth.

Fig. S2. The *hdl* mutant exhibits a loss of apical dominance phenotype.

Fig. S3. The *hdl* mutant plants occasionally show defects in the initiation of leaflets.

Fig. S4. Genetic analysis of *hdl-1* and *hdl-2* mutants.

Fig. S5. Complementation of the *hdl* mutant with *p35S::HDL.*

Fig. S6. Phylogenetic analysis of WOX family proteins in Arabidopsis and *M. truncatula*.

Fig. S7. Sequence alignment of HDL and Arabidopsis WUSCHEL.

Fig. S8. *In situ* hybridization analysis using the control sense *HDL* probe.

Fig. S9. Transcript abundance of endogenous *HDL* and exogenous *HDL-VP64* in *p35S::HDL-VP64* transgenic plants.

Fig. S10. Subcellular localization of the mutated HDL proteins.

Table S1. Primers used in this study.

Supplementary Figures S1-S10 and Table S1
