# Supplementary material for: HEADLESS, a WUSCHEL homolog, uncovers novel aspects of shoot meristem regulation and leaf blade development in Medicago truncatula
Source: J Exp Bot. 2018 Sep 29;70(1):149–63. doi: 10.1093/jxb/ery346 (PMC6305195; doi:10.1093/jxb/ery346)
Supplement: Supplementary Figures S1-S10 and Table S1 [file ery346_suppl_supplementary_figures_s1-s10_table_s1.pdf]

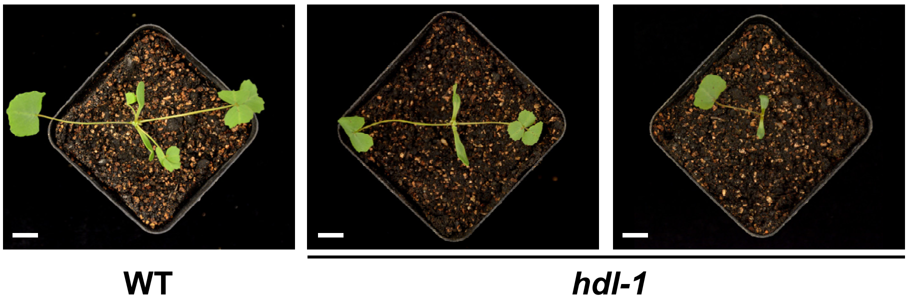

Fig. S1 The *hdl* mutant shows a defect in leaf outgrowth. *hdl-1* mutant seedlings (13 DAG) with increasing severity of defects affecting leaf outgrowth, and a wild-type seedling for comparison. Bars = 1 cm.

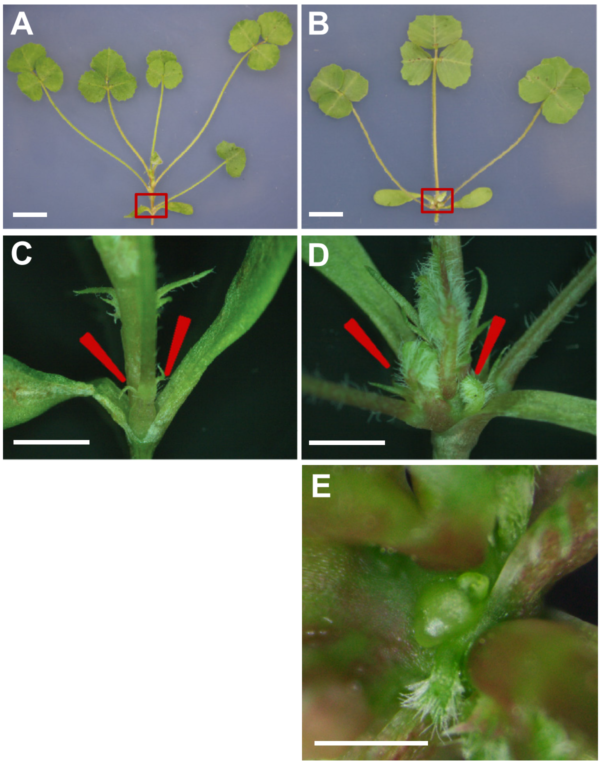

Fig. S2 The *hdl* mutant exhibits a loss of apical dominance phenotype. (A, B) The phenotype of the wild type (A) and *hdl-1* mutant (B) at seedling stage. Bars = 1 cm. (C, D) The magnifications of indicated regions in (A) and (B), respectively. Arrows indicates outgrown leaves at the axil of cotyledon in *hdl-1*, which is absent in the wild type. Bars = 1 cm. (E) Several dome-shaped structure forms among petiole base in *hdl-1*. Bar = 1 mm.

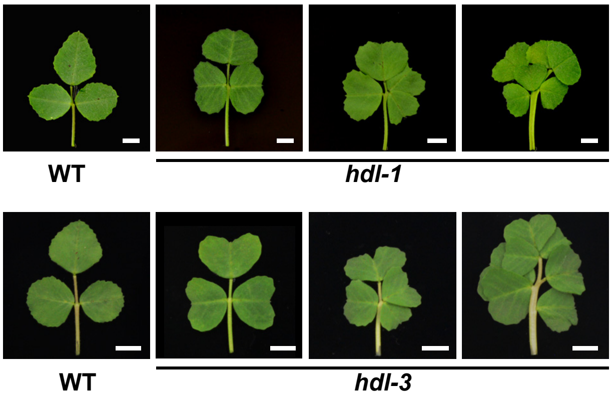

Fig. S3 The *hdl* mutant plants occasionally show defects in the initiation of leaflets. The *hdl* leaves occasionally show extra leaflets in contrast to the wild type. Bars = 0.5 cm.

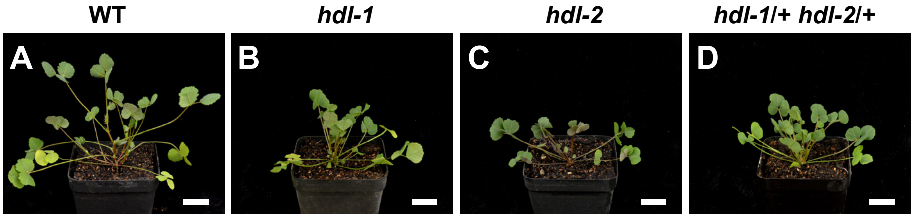

Fig. S4 Genetic analysis of *hdl-1* and *hdl-2* mutants. Phenotypes of the wild type (A), *hdl-1* (B), *hdl-2* (C) and the *hdl-1*/+ *hdl-2*/+ F1 progeny (D). Bars = 2 cm.

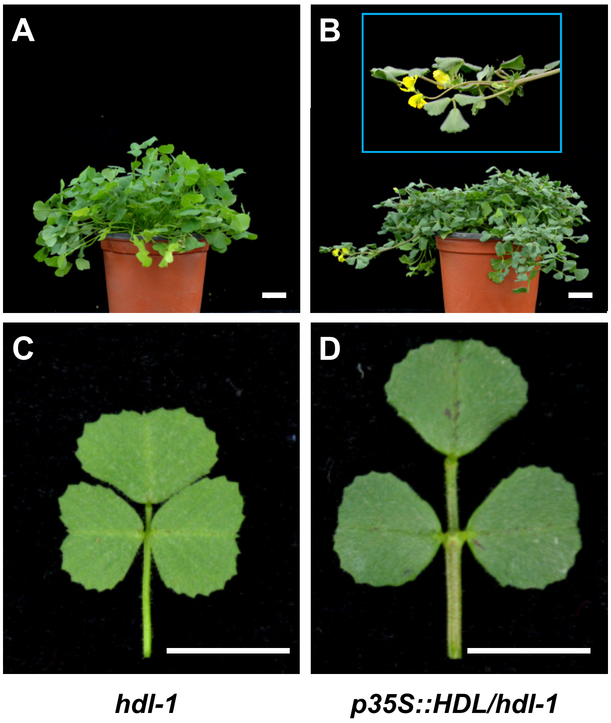

Fig. S5 Complementation of the *hdl* mutant with *p35S::HDL*. (A, B) Comparison of the *hdl-1* mutant (A) and *hdl-1* complemented with *p35S::HDL* (B). Bars = 2 cm. (C, D) Leaf phenotypes of the *hdl-1* mutant (C) and *hdl-1* complemented with *p35S::HDL* construct (D). Bars = 1 cm.

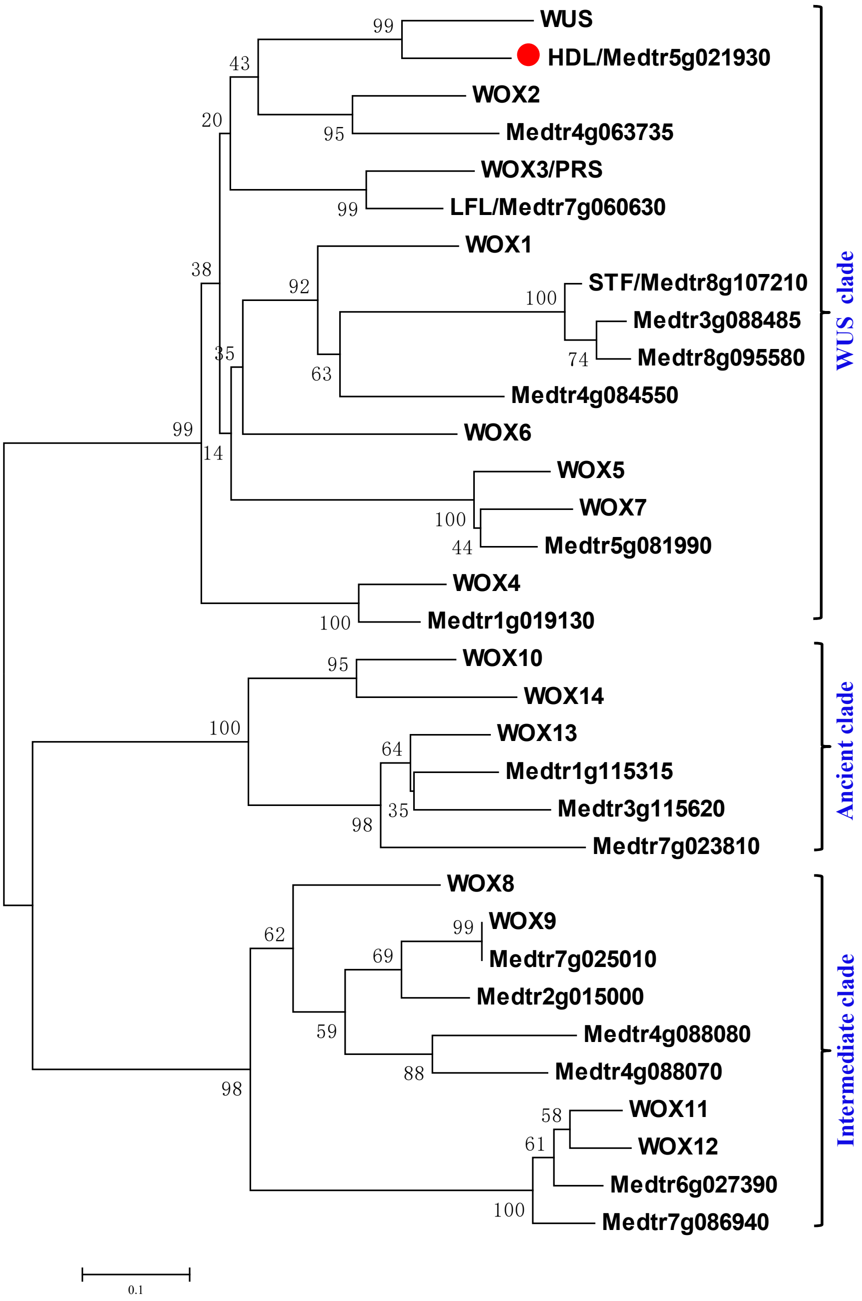

Fig. S6 Phylogenetic analysis of WOX family proteins in Arabidopsis and *M. truncatula*. Full-length amino acid sequences were aligned using ClustalW and the tree was constructed using MEGA4 with 1000 replicates to generate bootstrap values.

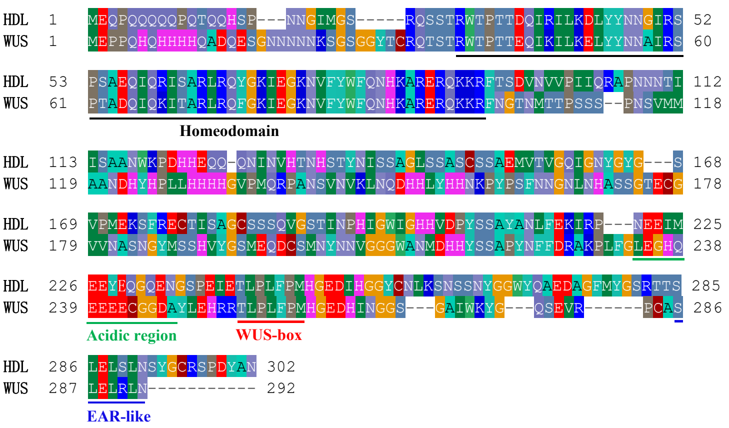

Fig. S7 Sequence alignment of HDL and Arabidopsis WUS. The conserved homeodomain, acidic region, WUS-box and EAR-like motif sequences are underlined in black, green, red and blue, respectively.

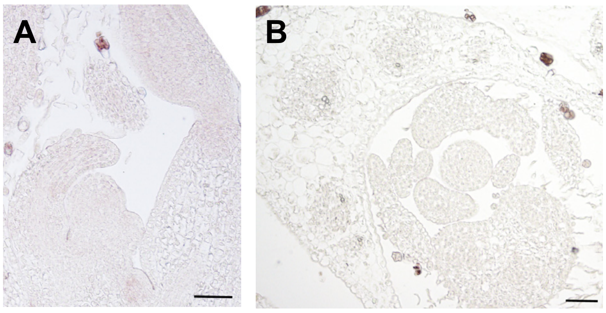

Fig. S8 *In situ* hybridization analysis using the control sense *HDL* probe. (A) *In situ* hybridization of *HDL* sense probe in the longitudinal section of the vegetative shoot apex. Bar = 50 µm. (B) *In situ* hybridization of *HDL* sense probe in the cross section of the vegetative shoot apex. Bar = 50 µm.

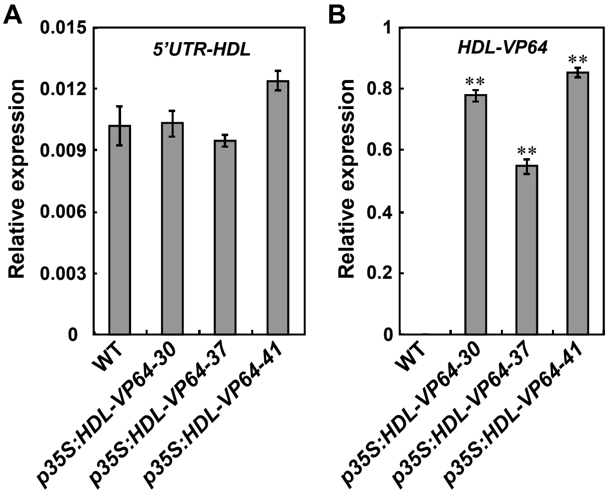

Fig. S9 Transcript abundance of endogenous *HDL* and exogenous *HDL-VP64* in *p35S::HDL-VP64* transgenic plants. (A) Transcript levels of endogenous *HDL* in the shoot apices of wild type and *p35S::HDL-VP64* transgenic plants. (B) Transcript abundance of exogenous *HDL-VP64* in the shoot apices of wild type and *p35S::HDL-VP64* transgenic plants. Error bars represent the standard error of three replicate experiments. The asterisks indicate significant differences (** $p < 0.01$, Student *t*-test) compared with the wild type.

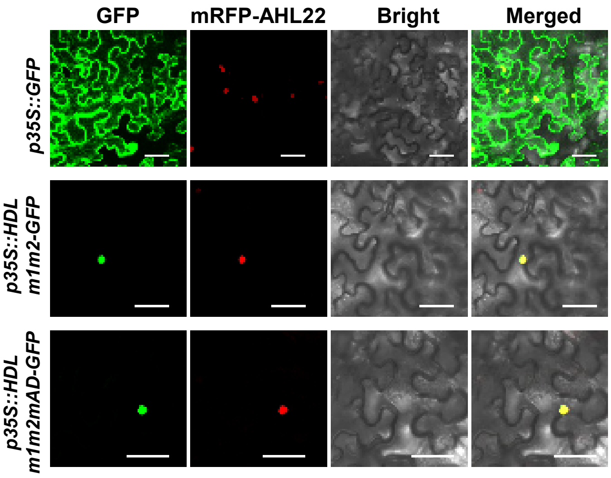

Fig. S10 Subcellular localization of the mutated HDL proteins. Subcellular localization of *p35S::GFP*, *p35S::HDLm1m2-GFP* and *p35S::HDLm1m2mAD-GFP* in tobacco epidermal cells. Nuclear protein AHL22 was used as a nuclear marker. Bars = 50 µm.

Table S1 Primers used in this study.

Primer name	Sequence (5'→3')
**For Overexpression**	
cacc-HDL-F	caccATGGAACAGCCTCAACAACAACAA
HDL-R	TTAATTAGCATAATCTGGTGACCTACA
HDL-no SC-R	ATTAGCATAATCTGGTGACCTACA
HDL-cVP64-R	GCGCGTCAGAACCATTAGCATAATCTGGTGAC
cVP64-HDL-F	TGCTAATGGTTCTGACGCGCTGGACGATTTC
cVP64-R	TTATAACATATCGAGATCG
**For Complementation**	
HDL-pro-*Kpn*I-iF	attcgagctcggtaccCACATTTTTGCACAATTTTATTAAAAAATACAAAA
HDL-1366ASC-*Pst*I-iR	ttgtatatcactgcagCCAAACTTGCCGTACAAGTTGTCAGTTCCT
**For Genotyping**	
HDL-216F	GCTCTCTTTTGTCCCTTCCC
HDL-862R	TCAGGTTTCCAATTAGCAGCA
HDL-989F	GCAGCAGTCAAGTTGGAAGT
HDL-R	TTAATTAGCATAATCTGGTGACCTACA
STF-F	ATGTGGATGGTGGGTTACAATG
STF-R	TCAGTTTTTCAAGGGAAGAAACT
LRT31-F	CTCCTCTCGGGGTCGTGGTT
LTR6-R	GCTACCAACCAAACCAAGTCAA
**For RT-PCR**	
HDL-F	ACACTCACCTACCCTCTCTA
HDL-R	TTAATTAGCATAATCTGGTGACCTACA
MtActin-RT-F	TCTTACTCTCAAGTACCCCATTGAGC
MtActin-RT-R	GTGGGAGTGCATAACCCTCATAGATT
**For *in situ* Hybridization**	
HDL-IS-F	ATGGAACAGCCTCAACAACA
HDL-IS-R	AATTAATACGACTCACTATAGGGTTAATTAGCATAATCTGGTG
**For qRT-PCR**	
Medtr4g106590-qF	AGTGAAGGATGGCGAGAAATG
Medtr4g106590-qR	AGGATAATGTTGATGGTGAGAGTG
Medtr3g078613-qF	TGTCACCGTCTGCAATGTCG
Medtr3g078613-qR	TCCTCTTCCTCAGTCCTAATCC
Medtr1g049100-qF	CAACAAACCAAAGCTAGAAGAAGC
Medtr1g049100-qR	TCACGCTGGATACAGACAGTG
Medtr5g036480-qF	TCACCCGACGTTCTTGATTC
Medtr5g036480-qR	AACATCAAAAGAGGTAAACAAGTAAATG
Medtr3g093860-qF	GAAGTTCCTGTTGTGATCATGTC
Medtr3g093860-qR	CAGATTGTTTGACAGGCTTCG
Medtr3g088630-qF	GATGTTCCAGTTGTGGTTATGTC
Medtr3g088630-qR	GCAAATCTGATAACTGAAGAGGC
Medtr7g490310-qF	CACAGACACAGTTACAAGCAC

Medtr7g490310-qR	GGTCTTGTTCTGTCAGTCTCAG
Medtr4g051330-qF	ATCCTCTACAGCCAACTACCT
Medtr4g051330-qR	CATCCACTGAAGCATTAAATTTGAAG
Medtr3g015490-qF	ACACTCTTCTATTTCCCTTATTATCCTC
Medtr3g015490-qR	AACATTCTGTCAATAACACTGTCATC
Medtr8g038620-qF	TCCTGAAACCAGTTCAACTATCAG
Medtr8g038620-qR	TTGACTTTCTTCCATACCCTTCC
Medtr6g007460-qF	CAAACAACCCATCAACAATAAAAGG
Medtr6g007460-qR	ATTAAGAGAGCCATTCAACAAAATCT
HDL-qF	GCTGAGATGGTTACTGTAGGC
HDL-qR	ATGTGAGGGTTTATGGTACTTCC
STF-qF	CAGAATCACAAAGCAAGAGAAAGG
STF-qR	TCAAACACTGTCCTACTTGCG
HDL-UTR-qF	GCAGTAGTACTCTCTTTCACACTCACC
HDL-CDS-qR	TTGTGTCTGTGGTTGTTGTTGTTG
HDL-CDS-qF	CCTTGGAACTTAGCCTCAACTCTTA
VP64-qR	CCCAACATATCCAGGTCAAAGTC
MtAS2-qF	TGGAATTGGGAGCAACTATG
MtAS2-qR	TATTACCTATAGCATTAGAAACCCT
MtActin-qF	TCAATGTGCCTGCCATGTATGT
MtActin-qR	ACTCACACCGTCACCAGAATCC
**For Y2H and BiFC Assay**	
HDL-m1-F	AAATGGTTCACCTGAAATTGAAACCGCCCCTGCGTTCCCTATGCAT GGTGAAGACATTC
HDL-m1-R	GAATGTCTTCACCATGCATAGGGAACGCAGGGGCGGTTTCAATTT CAGGTGAACCATTT
HDL-m2-F	GTTCTCGTACTACTTCCTTGGAAGCGAGCGCCAACTCTTACGGCT GTAGGTCAC
HDL-m2-R	GTGACCTACAGCCGTAAGAGTTGGCGCTCGCTTCCAAGGAAGTAG TACGAGAAC
HDL-mAD-F	CAACTTATTTGAAAAAATAAGACCAAATGCAGCAATCATGGCAGC ATATGCACAAGGACAAGCAAATGGTTCACCTGAAA
HDL-mAD-R	TTTCAGGTGAACCATTTGCTTGTCCTTGTGCATATGCTGCCATGATT GCTGCATTTGGTCTTATTTTTTCAAATAAGTTG
MtTPL-attB-F	CAAAAAAGCAGGCTTCATGTCATCTCTGAGTAGGGAATTG
MtTPL-attB-R	CAAGAAAGCTGGGTCTCATCTTTGTGCTTGGTCTG
attB adaptor-F	GTGGGGACAAGTTTGTACAAAAAAGCAGGCTTC
attB adaptor-R	GTGGGGACCACTTTGTACAAGAAAGCTGGGTC
**For Luciferase Assay**	
caccGXLBD-F	caccATGAAGCTACTGTCTTCTATCG
HDL-R	TTAATTAGCATAATCTGGTGACCTACA
cVP64-R	TTATAACATATCGAGATCG
